# A dual compartment cuvette system for correcting scattering in whole-cell absorbance spectroscopy of photosynthetic microorganisms

**DOI:** 10.1007/s11120-021-00866-8

**Published:** 2021-08-14

**Authors:** John R. D. Hervey, Paolo Bombelli, David J. Lea-Smith, Alan K. Hulme, Nathan R. Hulme, Atvinder K. Rullay, Robert Keighley, Christopher J. Howe

**Affiliations:** 1 Department of Biochemistry, University of Cambridge, Hopkins Building, Downing Site, Tennis Court Road, Cambridge, CB2 1QW UK; 2 School of Biological Sciences, University of East Anglia, Norwich Research Park, Norwich, NR4 7TJ UK; 3 Starna Scientific Ltd, Hainault Business Park, 52/54 Fowler Rd, Ilford, IG6 3UT UK; 4 Shimadzu UK Limited, Unit 1, Mill Crt, Featherstone, MK12 5RD UK

**Keywords:** Absorption spectroscopy, Cyanobacteria, Microalgae, Light scattering, Chromophores, Whole cell spectra

## Abstract

Absorption spectroscopy is widely used to determine absorption and transmission spectra of chromophores in solution, in addition to suspensions of particles, including micro-organisms. Light scattering, caused by photons deflected from part or all of the cells or other particles in suspension, results in distortions to the absorption spectra, lost information and poor resolution. A spectrophotometer with an integrating sphere may be used to alleviate this problem. However, these instruments are not universally available in biology laboratories, for reasons such as cost. Here, we describe a novel, rapid, and inexpensive technique that minimises the effect of light scattering when performing whole-cell spectroscopy. This method involves using a custom made dual compartment cuvette containing titanium dioxide in one chamber as a scattering agent. Measurements were conducted of a range of different photosynthetic micro-organisms of varying cell size and morphology, including cyanobacteria, eukaryotic microalgae and a purple non-sulphur bacterium. A concentration of 1 mg ml^−1^ titanium dioxide, using a spectrophotometer with a slit width of 5 nm, produced spectra for cyanobacteria and microalgae similar (1–4% difference) to those obtained using an integrating sphere. The spectrum > 520 nm was similar to that with an integrating sphere with the purple non-sulphur bacterium. This system produced superior results to those obtained using a recently reported method, the application of the diffusing agent, Scotch™ Magic tape, to the side of the cuvette. The protocol can be completed in an equivalent period of time to standard whole-cell absorbance spectroscopy techniques, and is, in principle, suitable for any dual-beam spectrophotometer.

**Supplementary Information:**

The online version contains supplementary material available at 10.1007/s11120-021-00866-8.

## Introduction

Optical absorption spectroscopy is a technique used to determine how solutions and particles in suspension interact with specific wavelengths of light. It is widely applied to a range of biological and chemical suspensions and is especially useful in determining the pigment composition of photosynthetic organisms (Merzlyak et al. 2008). However, the apparent absorbance of a suspension at a given wavelength depends not only on the actual absorbance but also on the scattering of light by particles and structures present in the suspension (Castanho et al. 1997; Latimer and Eubanks 1962; Merzlyak et al. 2008; Merzlyak and Naqvi 2000; Naqvi et al. 2004; Prado et al. 1996; Twersky 1970). The consequences of scattering are complex and depend on the specific wavelength examined, and the size, shape, and structure of the individual particles in suspension (Latimer and Eubanks 1962; Merzlyak et al. 2008; Ritchie and Sma-Air 2020a; Twersky 1970). This distorts the absorption measurements, as a portion of the signal recorded will be due to the variable scattering effect, rather than the absorption of light (Latimer and Eubanks 1962; Merzlyak et al. 2008; Merzlyak and Naqvi 2000; Naqvi et al. 2004; Twersky 1970). This problem has been widely studied, and various methods have been proposed to correct for the light scattered (Jackson et al. 2014; Latimer and Eubanks 1962; Merzlyak and Naqvi 2000; Naqvi et al. 2004; Shibata et al. 1954; Smith et al. 1957). Ideally, an integrating sphere is used, in order to collect all of the scattered light at the detector (Latimer and Eubanks 1962; Merzlyak and Naqvi 2000; Ritchie and Sma-Air 2020a, b). Spectrophotometers incorporating integrating sphere detectors are specialised pieces of equipment and too costly for many research laboratories. Therefore, other techniques to correct for scattering have been developed. Commonly, these utilise a diffuser on the front of the cuvette to scatter uniformly the beam coming from the light source (Jackson et al. 2014; Shibata et al. 1954; Smith et al. 1957). Scattering introduced by the diffuser is much greater than the scattering from the sample, so the scattering from the sample becomes negligible and is no longer apparent in the recorded spectra. In 1954, Shibata et al. (1954) proposed the use of filter paper dipped in paraffin wax as a diffuser and in 1957, Smith et al. refined the method by exchanging the waxed paper for opalescent glass (Smith et al. 1957). More recently, opaque tape such as Scotch™ Magic tape has been used as a diffuser (Jackson et al. 2014). However, many of these diffusing agents are not easily standardised, so results may vary from experiment to experiment, and from lab to lab; this makes reporting and reproduction of results difficult.

**Fig. 1:**
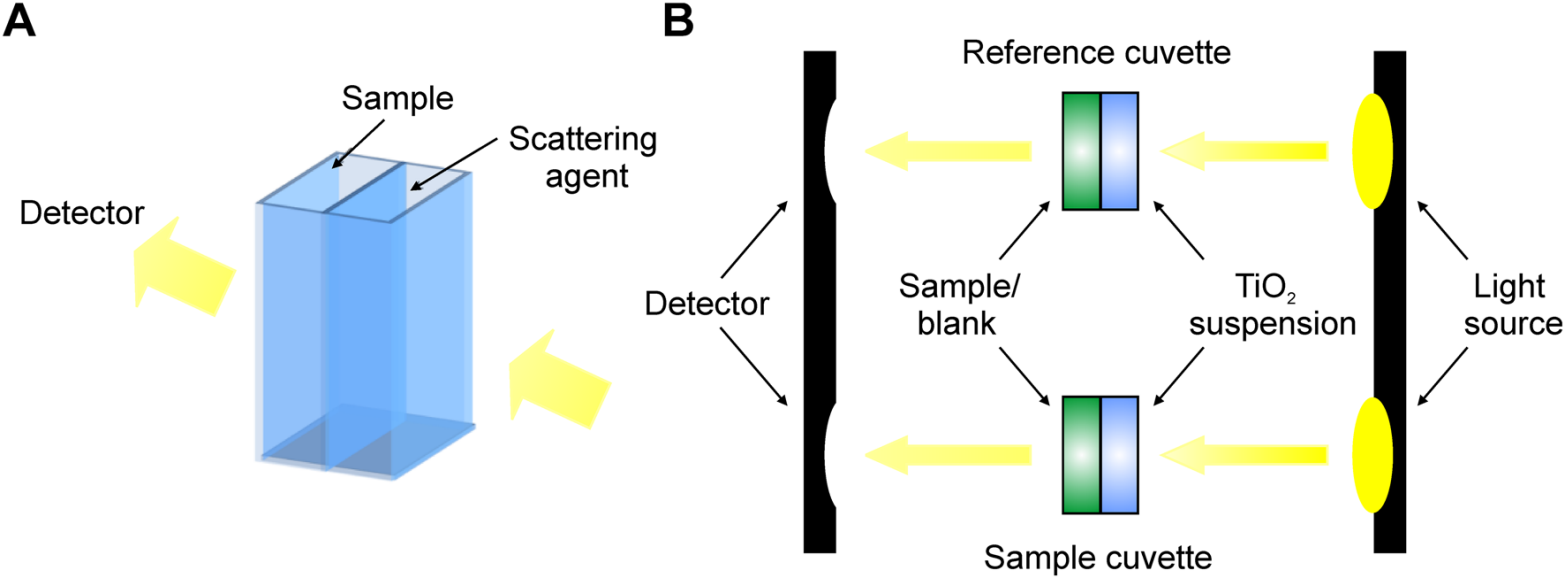
Design and use of the custom, two chamber cuvette. **a** Diagram of the device detailing the compartments for inclusion of a sample and a scattering agent; **b** Diagram detailing use of the cuvette in a dual-beam spectrophotometer

In this study, we outline a new diffuser system for the correction of scattering in biological suspensions, that is easily standardised, inexpensive, and produces accurate and reproducible results comparable to data obtained using an integrating sphere. This technique utilises custom built dual-compartment cuvettes, manufactured by Starna Scientific, in which scattering is corrected via a suspension of titanium dioxide. These cuvettes can be used in standard dual beam spectrophotometers, making this technique accessible to the majority of biological laboratories. Figure 1 depicts the details of the dual-compartment cuvettes and their use in a dual-beam spectrophotometer. Titanium dioxide is used, as it is widely available and inexpensive, and forms an opaque, scattering suspension in water. Importantly, titanium dioxide can be standardised with respect to particle size and concentration, thus making it suitable as an easily standardised and reproducible diffuser. We demonstrate that this system is superior to using Scotch™ Magic tape as the diffuser, producing results comparable to data collected using an integrating sphere detector.

## Materials and methods

### Culture medium and growth conditions

*Synechocystis* sp. PCC 6803 (*Synechocystis*) strains were cultured in BG11 medium at 30 °C. *Chlorella vulgaris* CCAP 211/52 (*Chlorella*) and *Chlamydomonas reinhardtii* CC 1021 (*Chlamydomonas*) strains were cultured in standard TP medium at 30 °C. *Synechococcus* sp. PCC 7002 (*Synechococcus*) and *Dunaliella salina* CCAP 19/12 (*Dunaliella*) were grown in artificial salt water medium (ASW) at 25 °C. *Rhodopseudomonas palustris* CGA009 (*Rhodopseudomonas*) was grown in a minimal salts medium, supplemented with 10 mM urea and 50 mM glycerol. All of these strains were cultured in an illuminated incubator with continuous shaking (125 rpm) and light (~ 50 µE m^−2^ s^−1^).

### Spectrophotometric methods

One set of spectra was recorded in a Shimadzu UV-2600 spectrophotometer, with a slit width of 5 nm. Reference spectra were recorded using the same device with an add-on ISR-2600Plus two-detector integrating sphere, with a slit width of 5 nm. A second set of data were collected using a Shimadzu UV-1800 spectrophotometer, which has a slit width of 1 nm. These data were compared to reference spectra collected in the Shimadzu UV-2600 with the add-on ISR-2600Plus two-detector integrating sphere, with the slit width set to 1 nm. All experiments were conducted at room temperature.

### Dual compartment cuvette measurements with titanium dioxide

Two quartz cuvettes were used in the dual-beam spectrophotometer. In these customized cuvettes, the chamber is divided into two compartments, each with an optical path of 5 mm (Fig. 1). The cuvettes were orientated so that the investigation beam passed through both chambers. A baseline reading was taken with blank growth medium in one chamber of both cuvettes (i.e., the sample and the reference cuvettes) and a premixed suspension of titanium dioxide (Sigma-Aldrich, Titanium IV oxide, anatase, -325 mesh) in water in the other chamber (Fig. 1). The titanium dioxide suspension was placed in the chamber closer to the light source, such that the beam passed through the suspension before passing through the sample. The spectrophotometer was zeroed with this setup by recording a baseline correction. The cuvette was inverted several times each time a new sample was measured, to ensure that the titanium dioxide remained in suspension and did not settle. The concentrations of titanium dioxide used were 0.1, 0.2, 0.5 and 1 mg ml^−1^. To record a spectrum, the titanium dioxide suspension was left in place in both cuvettes, but the blank medium in the sample cuvette was replaced with the sample (e.g., microalgal cell suspension). For the reference spectra recorded using the integrating sphere detector, the titanium dioxide suspension was replaced with water. This way the path length of the sample was the same as that used for the other samples. Three technical replicate measurements were taken for each sample.

### Single compartment cuvette measurements with Scotch™ Magic tape

Two standard, single-compartment, quartz cuvettes were used in the dual-beam spectrophotometer. These cuvettes had 0, 1, 5, or 10 layers of Scotch™ Magic tape applied to the side of the cuvette that was closer to the light source, such that the investigation beam passed through the tape before passing through the sample. Adding more than 10 layers resulted in a cuvette unable to fit in the spectrophotometer. Prior to analysis of samples, the spectrophotometer was zeroed, and a baseline recorded, with blank medium in both cuvettes. To record a spectrum, the blank medium in the sample cuvette was replaced with the sample (e.g., microalgal cell suspension). For the reference spectra recorded using the integrating sphere detector, the tape was removed from the cuvettes. Three technical replicate measurements were taken for each sample.

## Results

To determine the absorption profile of a range of photosynthetic organisms we analysed two cyanobacterial species (*Synechocystis* and *Synechococcus*), a non-sulphur purple bacterium (*Rhodopseudomonas*) and three eukaryotic microalgal species (*Chlorella*, *Chlamydomonas*, *Dunaliella*). Species were selected based on differences in size and shape, which are outlined in Table 1. In addition, a *Synechocystis* (‘Olive’) mutant lacking the phycocyanin portion (λ_max_ = 625 nm) of the light harvesting phycobilisome complex and whose cells are smaller than wild-type (Lea-Smith et al. 2014), and a cell wall deficient *Chlamydomonas* strain (CW15) (Davies and Plaskitt 1971), were also tested.

**Table 1:** Species examined in this study

Species	Shape	Size (µm)	References	OD660	OD730	OD750
*Synechocystis* sp. PCC 6803	Spherical	2.02–2.06	Hayashi et al. (1982) and Lea-Smith et al. (2014)	0.75	0.61	0.57
*Synechocystis* sp. PCC 6803- Olive	Spherical	1.82	Lea-Smith et al. (2014)	0.33	0.27	0.25
*Synechococcus* sp. PCC 7002	Ovoid	2.30/1.61	Lea-Smith et al. (2016)	0.38	0.30	0.28
*Chlorella vulgaris* CCAP 211/52	Spherical	3	le Grooth et al. (1985)	0.74	0.73	0.69
*Dunaliella salina* CCAP 19/12	Ovoid	5–10	Preetha et al. (2012)	0.66	0.65	0.64
*Chlamydomonas reinhardtii* CC1021	Spherical	10	Ratcliff et al. (2013)	0.19	0.20	0.20
*Chlamydomonas reinhardtii* CW15	Spherical	10	Davies and Plaskitt (1971) and Ratcliff et al. (2013)	0.34	0.38	0.37
*Rhodopseudomonas palustris* CGA009	Rod	2/0.5	van Niel (1944)	0.35	0.29	0.28

Size refers to the diameter in spherical cells and length/width in ovoid and rod shaped cells. Optical density is given at time of measurement at standard wavelengths

### Analysis of the absorption spectra of strains using the dual compartment cuvette with titanium dioxide

**Fig. 2:**
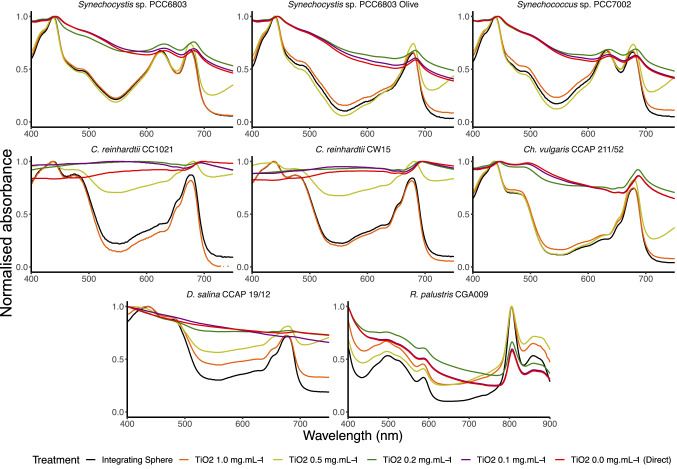
Comparison of whole-cell absorbance spectra with the dual compartment cuvette (slit width 5 nm). Samples were analysed using the integrating sphere (black) or in the dual compartment cuvette with 0 (red), 0.1 (purple), 0.2 (green), 0.5 (yellow) or 1 (orange) mg ml^−1^ TiO_2_. Results are standardised as described in the text. The mean of three samples is displayed

We first analysed the absorption profile of all the strains using a spectrophotometer with an integrating sphere detector and a slit width of 5 nm. The same samples were then examined using the dual chamber cuvette system with no titanium dioxide or different concentrations between 0.1 and 1 mg ml^−1^. The raw data (Fig. S1) were analysed in the region 400–750 nm, except for *R. palustris* where the range was from 400 to 900 nm. Each spectrum was normalised such that the maximum absorbance value recorded was made equal to 1. This was achieved by dividing every point of the curve by the maximum absorbance value measured in the considered interval. This normalisation permits a direct comparison of spectra derived from samples with various cell densities (Fig. 2).

When titanium dioxide at 1 mg ml^−1^ was used, the spectra of each of the cyanobacterial and microalgal species were similar to the profile obtained using the integrating sphere, in terms of both the magnitude and the overall shape. When titanium dioxide at 0.5 mg ml^−1^ was used, the spectral profiles were similar from 400 to 680 nm but diverged between 680 and 750 nm. (The spectral profiles were less similar for the *C. reinhardtii* strains.) This concentration is therefore unsuitable for most applications since this part of the spectrum includes absorption from chlorophyll *a*. The *Rhodopseudomonas* profile (Fig. 2) was similar between the results obtained using the integrating sphere and the dual chamber cuvette system when titanium dioxide was used at 1 mg ml^−1^, except below < 520 and > 920 nm. Therefore, this technique would be suitable for analysing absorption of the main pigments in this species in the infrared part of the spectrum, specifically bacteriochlorophyll *a* (λ_max_ = 803 nm) and *b* (λ_max_ = 860 nm) (Merzlyak et al. 2008). Optical density of the culture did not appear to have a noticeable effect on the optimum concentration of TiO_2_. Table 1 shows the optical density of each culture, at standard wavelengths, and includes a range from 0.2 to 0.8, which covers the standard range that would reasonably be used in these types of measurements.

**Fig. 3:**
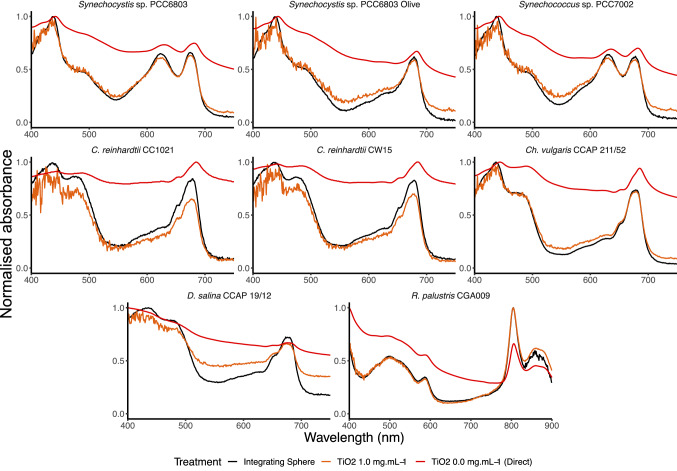
Comparison of whole-cell absorbance spectra with the dual compartment cuvette (slit width 1 nm). Samples were analysed using the integrating sphere (black) or in the dual compartment cuvette with 0 (red) or 1 (orange) mg ml^−1^ TiO_2_. Results are standardised as described in the text. The mean of three samples is displayed

We then analysed all strains using a spectrophotometer with an integrating sphere detector and a slit width of 1 nm, followed by the dual chamber cuvette system with no titanium dioxide or 1 mg ml^−1^ (Fig. S2; Fig. 3). The spectral profiles of all strains were similar between those analysed using the integrating sphere and the dual chamber cuvette system with titanium dioxide at 1 mg ml^−1^. However, with the exception of *Rhodopseudomonas*, variation between replicates was higher between 400 and 500 nm compared to samples examined using the spectrophotometer with a slit width of 5 nm. This is likely to be due to an increase in noise as a result of the reduced radiant energy at these wavelengths, so the use of a slit width of 5 nm rather than 1 nm alleviates this concern.

### Analysis of the absorption spectra of strains using the single compartment cuvette with Scotch™ Magic tape

**Fig. 4:**
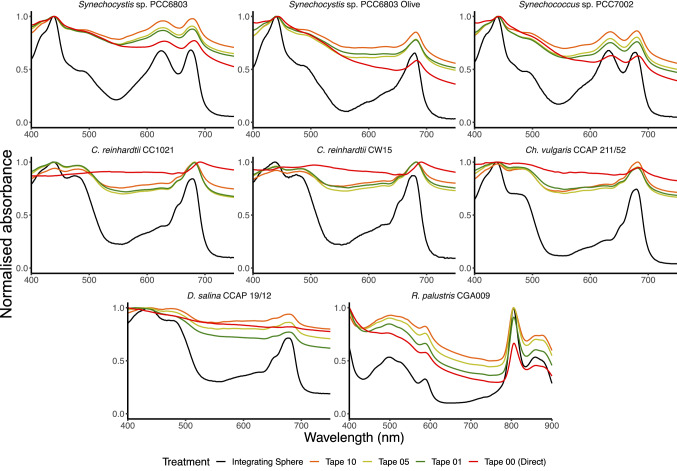
Comparison of whole-cell absorbance spectra with Scotch™ Magic tape (slit width 5 nm). Samples were analysed using the integrating sphere (black) or in the single compartment cuvette coated with 0 (red), 1 (green), 5 (yellow) or 10 (orange) pieces of Scotch™ Magic tape. Results are standardised as described in the text. The mean of three samples is displayed

**Fig. 5:**
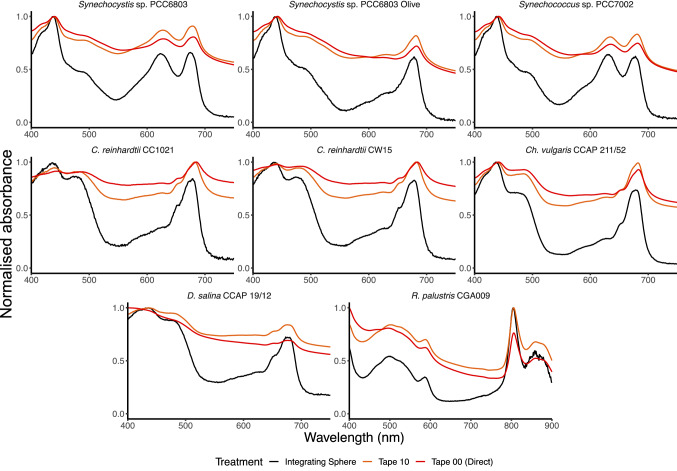
Comparison of whole-cell absorbance spectra with Scotch™ Magic tape (slit width 1 nm). Samples were analysed using the integrating sphere (black) or in the single compartment cuvette coated with 0 (red) or 10 (orange) pieces of Scotch™ Magic tape. Results are standardised as described in the text. The mean of three samples is displayed

Next we performed a comparison of this method with one previously reported, the coating of a single chamber cuvette with Scotch™ Magic tape (Jackson et al. 2014). The samples were analysed in a spectrophotometer with a slit width of 5 nm (Fig. S3; Fig. 4) or 1 nm (Fig. S4; Fig. 5), with different numbers of layers of Scotch™ Magic tape. With a slit width of 5 nm, adding even one layer of tape markedly changed the profile compared to analysing the sample using the cuvette only. However, the profile of none of the samples measured with either 1, 5 or 10 layers of tape resembled closely the profile across the spectrum obtained using the integrating sphere. Surprisingly, when using the spectrophotometer with the slit width of 1 nm, there was little difference in the profile of samples between the cuvette only and the cuvette with ten layers of tape, with the exception of *Dunaliella*.

### Analysis of absorbance is optimal using the dual compartment cuvette with 1 mg ml^−1^ titanium dioxide in a spectrophotometer with a slit width of 5 nm

**Fig. 6:**
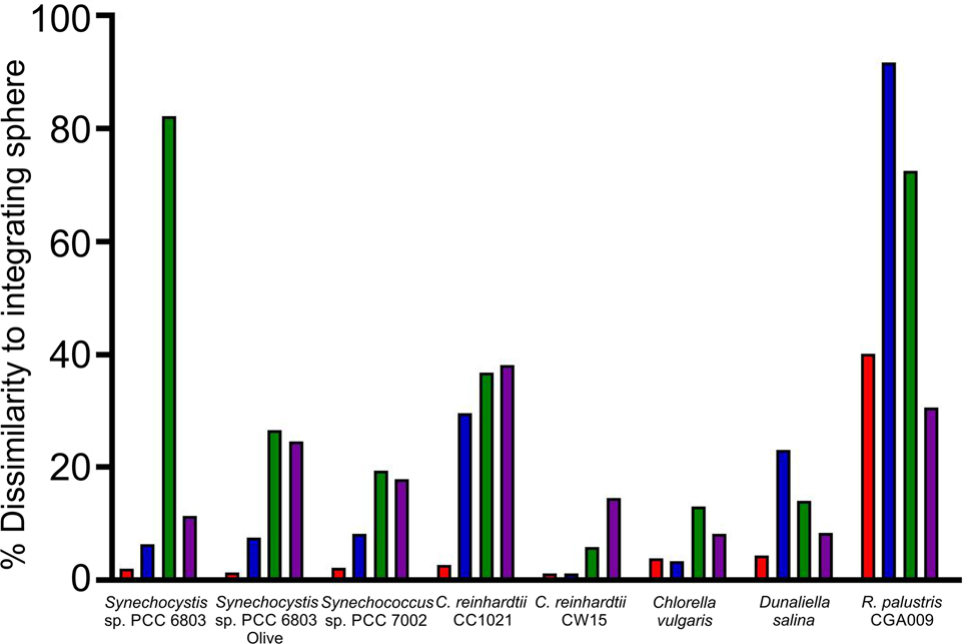
Average difference across the spectrum (400–750 nm (400–900 nm for *R. palustris*)) between data obtained using the integrating sphere and the dual compartment/TiO_2_ and single compartment/tape systems. The figure shows the result most similar to the integrating sphere from among the replicate experiments conducted using the dual compartment/TiO_2_ system with a slit width of 5 nm (red) and 1 nm (blue), and the single compartment/tape system with a slit width of 5 nm (green) and 1 nm (purple)

Finally, we determined the average difference across the spectrum (400–750 nm for cyanobacteria/microalgae; 400–900 nm for *Rhodopseudomonas*) between the different methods compared to the reference spectra collected with the integrating sphere (Fig. 6; Fig. S5). For all species, 1 mg ml^−1^ titanium dioxide in a dual compartment cuvette in a spectrophotometer with a slit width of 5 nm was the optimal method. Using a spectrophotometer with a slit width of 1 nm resulted in spectra more comparable to the reference than using a single compartment cuvette with layers of tape.

## Discussion

In this study, we tested seven microorganisms ranging in diameter from 2 to 10 µm using two different scattering agents. Overall, we showed that the two-compartment cuvette system with titanium dioxide can be used to obtain accurate absorption measurements, unhindered by the effects of light scattering, in standard dual-beam spectrophotometers, without an integrating sphere. This method should be easily replicated between different laboratories, since the concentration and particle size of the titanium dioxide are known and consistent. Increasing the concentration of titanium dioxide brought the measured spectra closer to the reference spectra in every case. Using a titanium dioxide concentration of 1 mg ml^−1^ resulted in spectra very similar to those collected using an integrating sphere, suggesting that this is the optimum concentration for the diffuser. Increasing the concentration of titanium dioxide further would not be beneficial, since the recorded spectra already matched the reference spectra almost perfectly, and the increased opacity from higher concentrations would only serve to reduce the signal that could be measured.

This method offers several advantages compared to those using waxed paper (Shibata et al. 1954), opalescent glass (Smith et al. 1957), or tape (Jackson et al. 2014) as the scattering diffuser. First, the spectra collected are similar to the reference spectra collected using the integrating sphere. Secondly, as previously mentioned, it is easily standardised and replicated across different laboratories and experiments. Thirdly, the method can be easily modified to change the scattering effect, by altering the concentration of titanium dioxide used. Finally, whilst customised cuvettes are needed, they can be used in any standard dual-beam spectrophotometer without modification, although a device with a slit width of 5 nm is optimal. Adding tape or other diffusers to the side of an existing cuvette add to its size, and can result in problems fitting the cuvette into the spectrophotometer. This method is therefore applicable to whole-cell spectroscopy of organisms and could potentially be applied to other opaque or suspended samples currently analysed using a spectrophotometer with an integrating sphere.

## Supplementary Information

Below is the link to the electronic supplementary material.

Supplementary file1 (PDF 1815 kb)

## Data Availability

The datasets generated during and/or analysed during the current study are available from the corresponding author on reasonable request.
