# Supplementary material for: A dual compartment cuvette system for correcting scattering in whole-cell absorbance spectroscopy of photosynthetic microorganisms
Source: Photosynth Res. 2021 Aug 14;151(1):61–9. doi: 10.1007/s11120-021-00866-8 (PMC8795073; doi:10.1007/s11120-021-00866-8)
Supplement: Supplementary file 1 — Supplementary file1 (PDF 1815 kb) [file 11120_2021_866_MOESM1_ESM.pdf]

Supplementary information

Figures S1-S5.

A dual compartment cuvette system for correcting scattering in whole-cell absorbance spectroscopy of photosynthetic microorganisms

John R. D. Hervey[1], Paolo Bombelli[1], David J. Lea-Smith[1,2], Alan K. Hulme[3], Nathan R. Hulme[3], Atvinder K. Rullay[4], Robert Keighley[4], Christopher J. Howe[1,*]
*Corresponding Author

[1]Department of Biochemistry, University of Cambridge, Hopkins Building, Downing Site, Tennis Court Road, Cambridge, CB2 1QW, United Kingdom.

[2]School of Biological Sciences, University of East Anglia, Norwich Research Park, Norwich, NR4 7TJ, United Kingdom.

[3]Starna Scientific Ltd, Hainault Business Park, 52/54 Fowler Rd, Ilford, IG6 3UT, United Kingdom.

[4]Shimadzu UK Limited, Unit 1, Mill Crt, Featherstone, MK12 5RD United Kingdom.

Corresponding author address: ch26@cam.ac.uk; Department of Biochemistry, University of Cambridge, Downing Site, Tennis Court Road, Cambridge, CB2 1QW, UK.

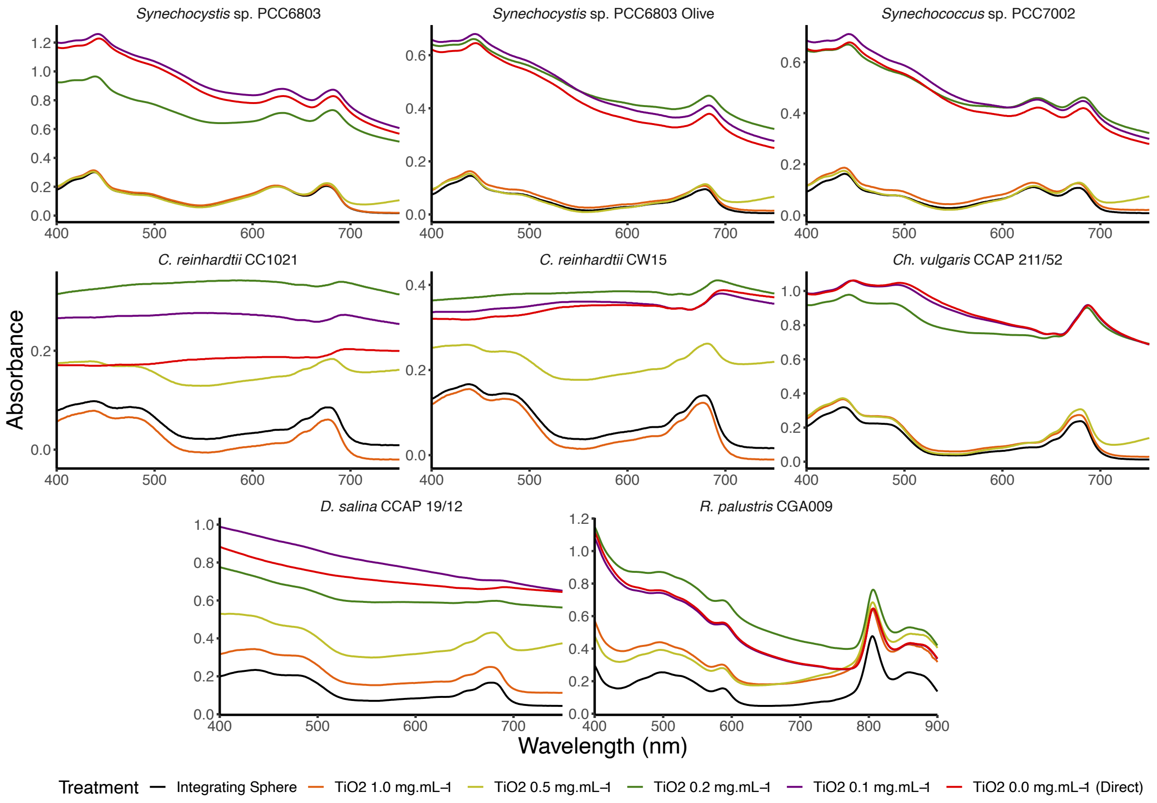

Fig. S1 Comparison of whole-cell absorbance spectra with the dual compartment cuvette (slit width 5 nm). Samples were analysed using the integrating sphere (black) or in the dual compartment cuvette with 0 (red), 0.1 (purple), 0.2 (green), 0.5 (yellow) or 1 (orange) mg.mL^{-1} TiO_2. Results are not standardised. The mean of three samples is displayed.

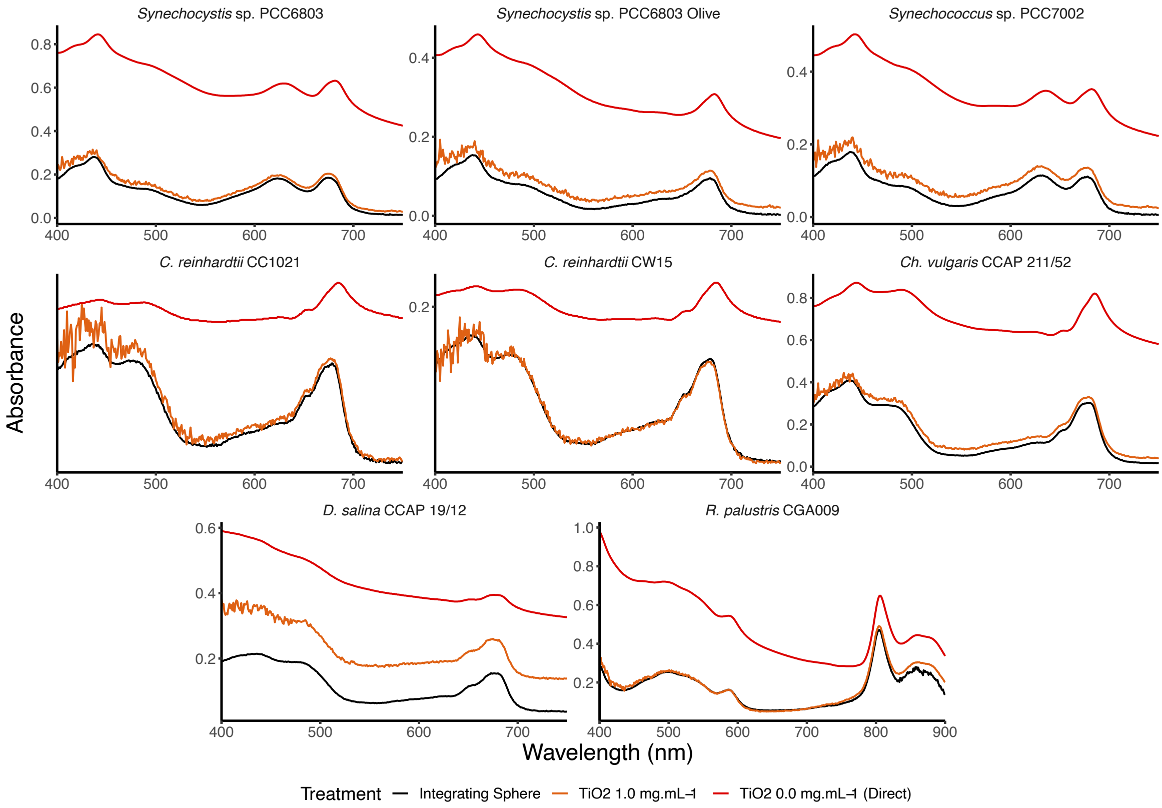

Fig. S2 Comparison of whole-cell absorbance spectra with the dual compartment cuvette (slit width 1 nm). Samples were analysed using the integrating sphere (black) or in the dual compartment cuvette with 0 (red) or 1 (orange) mg.mL^{-1} TiO_2. Results are not standardised. The mean of three samples is displayed.

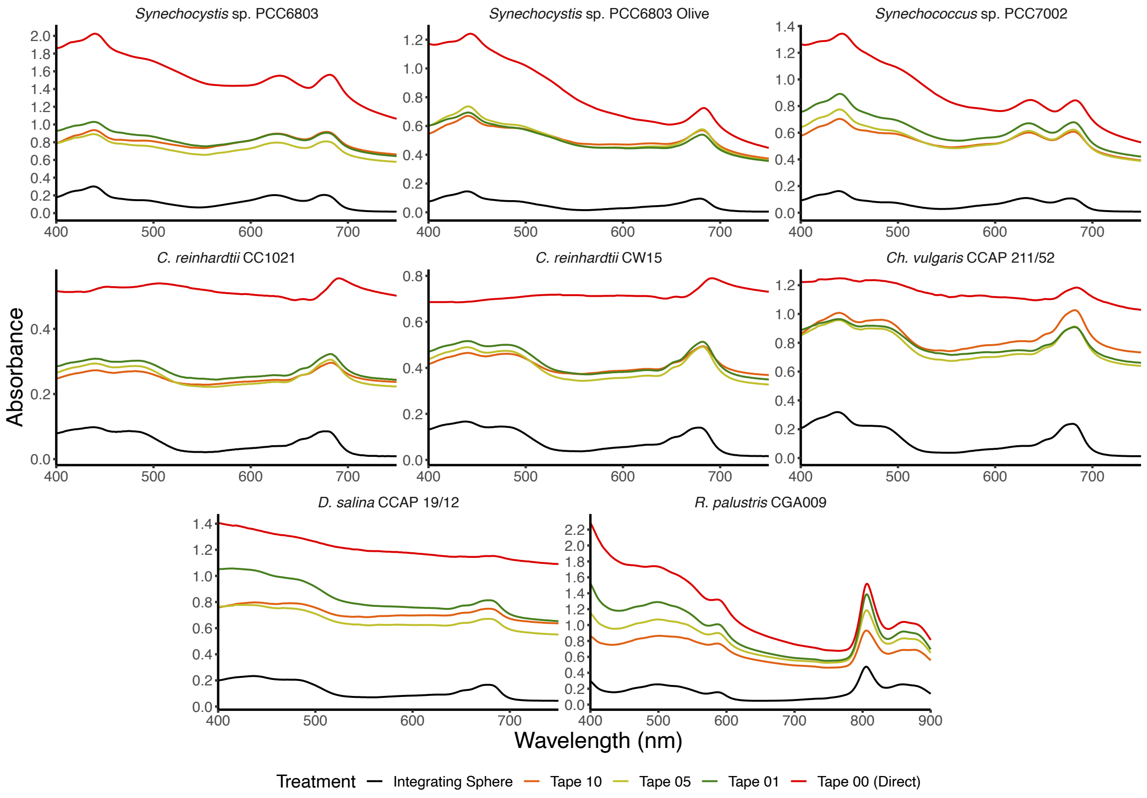

Fig. S3 Comparison of whole-cell absorbance spectra with Scotch™ Magic tape (slit width 5 nm). Samples were analysed using the integrating sphere (black) or in the single compartment cuvette coated with 0 (red), 1 (green), 5 (yellow) or 10 (orange) pieces of Scotch™ Magic tape. Results are not standardised. The mean of three samples is displayed.

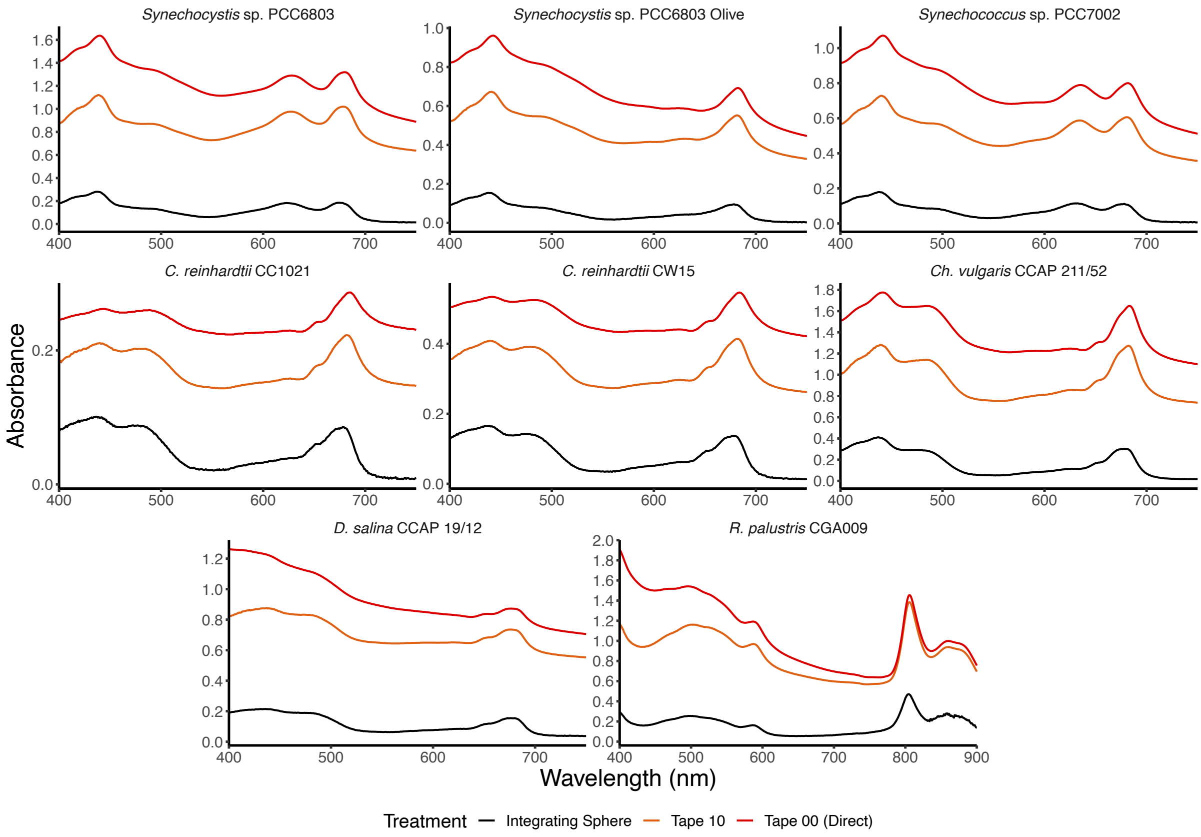

Fig. S4 Comparison of whole-cell absorbance spectra with Scotch™ Magic tape (slit width 1 nm). Samples were analysed using the integrating sphere (black) or in the single compartment cuvette coated with 0 (red) or 10 (orange) pieces of Scotch™ Magic tape. Results are not standardised. The mean of three samples is displayed.

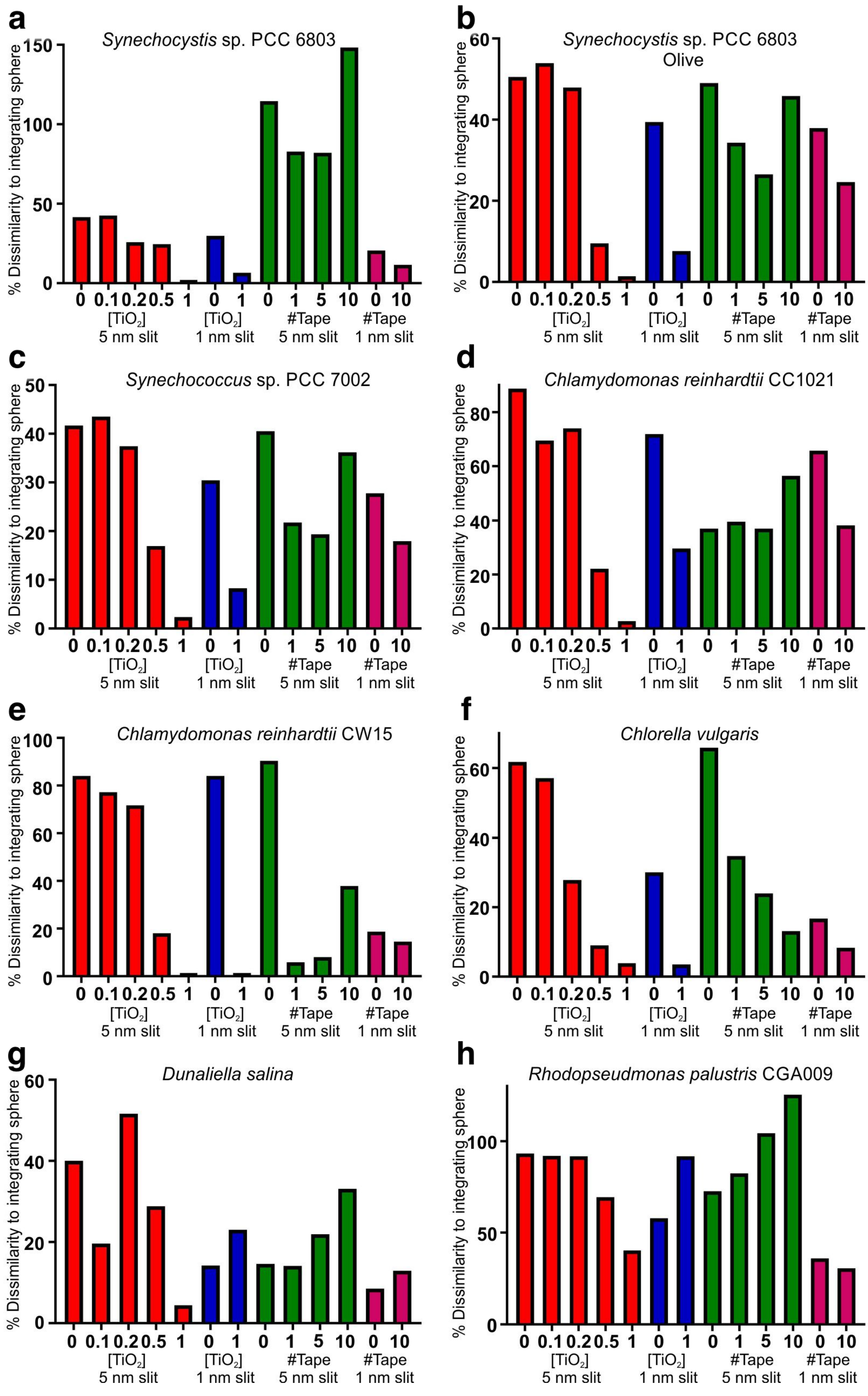

Fig. S5 Differences compared to results obtaining using the integrating sphere. Average differences between 400 - 750 nm (400 - 900 nm for *R. palustris*) results obtained using the four methods outlined in this paper compared to the optimal data acquired via the integrating sphere.
